# The Burden of Premature Mortality in Hamadan Province in 2006 and 2010 Using Standard Expected Years of Potential Life Lost: A Population-based Study

**DOI:** 10.4178/epih/e2012005

**Published:** 2012-08-31

**Authors:** Jalal Poorolajal, Nader Esmailnasab, Jamal Ahmadzadeh, Tahereh Azizi Motlagh

**Affiliations:** 1 Research Center for Health Sciences, Department of Epidemiology & Biostatistics, School of Public Health, Hamadan University of Medical Sciences, Hamadan, Iran.; 2 Department of Community Medicine, School of Medicine, Kurdistan University of Medical Sciences, Kurdistan, Iran.; 3 Department of Epidemiology & Biostatistics, School of Public Health, Hamadan University of Medical Sciences, Hamadan, Iran.; 4 Vice-Chancellor of Health Services, Hamadan University of Medical Sciences, Hamadan, Iran.

**Keywords:** Premature mortality, Life expectancy, Non-communicable diseases, Mortality, Iran

## Abstract

**OBJECTIVES:**

Examining the premature death rate represents the first step in estimating the overall burden of disease, reflecting a full picture of how different causes affect population health and providing a way of monitoring and evaluating population health. The present study was conducted to assess the burden of premature mortality in Hamadan Province, Iran in 2006 and 2010.

**METHODS:**

To calculate years of potential life lost (YPLL), the dataset was categorized into 5-year age groups based on each person's age at death. Then the age groups were subtracted from the relevant age-based life table produced by the World Health Organization in 2009. The YPLL for each individual were then added together to yield the total YPLL for all individuals in the population who died in a particular year. Finally, we calculated the YPLL for all sex-, age-, and cause-specific mortality rates and reported them as percentages.

**RESULTS:**

We analyzed 18,786 deaths, 9,127 of which occurred in 2006 and 9,659 in 2010. Mortality rates were higher in men than women for all age groups both in 2006 and 2010. In addition, age-specific mortality rates in both genders for all age groups were higher in 2010 than in 2006. The percentage of YPLL from ischemic heart diseases, cerebrovascular diseases, transport accidents, and intentional self-harm were among the greatest sources of premature death.

**CONCLUSION:**

The results of the present survey indicate that the eight major causes of premature death in both 2006 and 2010 were non-communicable diseases, especially ischemic heart diseases, cerebrovascular diseases, transport accidents, and intentional self-harm. Furthermore, our findings indicate a change in the role of non-communicable diseases in premature mortality in recent years.

## INTRODUCTION

Traditionally, mortality rates have frequently been used to estimate the extent of public health problems and to determine the relative importance of the different causes of death [1-3]. Although these rates play an important role in estimating health status, they often fail to account for the burden of premature mortality which is an important indicator of the health status of a population. In fact, since most deaths occur among those in older age groups, mortality rates are dominated by the underlying disease processes of the elderly [4].

Today, another mortality index, years of potential life lost (YPLL), is increasingly being used to set health priorities [5]. As a method, it is an alternative to death rates which gives more weight to deaths that occur among younger people. In other words, deaths among younger people contribute more to the YPLL measure than deaths among older people [6]. The YPLL concept estimates the average years a person would have lived if he or she had not died prematurely. This estimation inherently incorporates age at death, rather than merely the occurrence of death itself. This indicator is, therefore, a measure of premature mortality (early death) which is used to help quantify social and economic loss due to premature death [7]. To calculate YPLL, each person's age at death is subtracted from the reference age. In developed countries, such as the United States, the reference age is commonly set at 75, but it is essentially arbitrary [6].

Policy-makers face the challenge of responding to current disease prevention and control priorities. Such decisions should be based on summary measures that quantify the burden of disease at the population level [8], including premature mortality. Summary estimates such as YPLL serve as a common currency for reporting the burden of disease in certain populations. Measures of premature mortality reflect a full picture of how different causes affect population health and provide a way of monitoring and evaluating population health so that prevention and control actions can be taken rapidly when necessary [8].

In the developing countries inhabited by four-fifths of the world population, non-communicable diseases are fast replacing traditional communicable diseases in terms of impacting population health. By the year 2020, non-communicable diseases are expected to account for seven out of every ten deaths in developing countries compared to less than half that today. The so-called "epidemiological transition" poses serious challenges to health-care systems forced to make difficult decisions about the allocation of resources [9,10]. Therefore a measure of total mortality alone is not appropriate for gaining a full picture of the health status of a specific county or region. This gap has now been filled with YPLL, a comprehensive, internally consistent and comparable approach to determining current patterns of mortality. This new approach quantifies not only the number of deaths, but also the impact of premature death on a population.

Hamadan Province is situated in the north west of Iran and has a population of approximately 1.7 million people. This province had the highest mortality rate of the 31 provinces in the country in 2006 with a crude mortality rate of 5.6 per 1,000 population [11]. In addition, myocardial infarction, cerebrovascular accidents, and transport accidents were the first three major leading causes of death in Hamadan Province in 2004 [11]. This evidence indicates that special attention should be paid to this province in terms of mortality. Accordingly, the present study was conducted to assess the major causes of premature mortality in Hamadan Province in recent years.

## MATERIALS AND METHODS

This population-based study was conducted in Hamadan Province in 2011. The local Human Subject Review Board of Hamadan University of Medical Sciences approved the study. In the Islamic Republic of Iran, the District Health Departments (DHDs) are part of the District Health Network and are primarily responsible for preventive and primary health care services. The DHDs are the most peripheral functional unit of the District Health System and are responsible for collecting mortality data. Since 1998, a single uniform death certificate has been used throughout the country and is legally required to be issued by a physician for interment [12]. This form is compatible with the International Classification of Diseases Revision 10 (ICD-10) [13]. All physicians are trained on how to fill out this form as a part of their compulsory medical education. In special cases, death certificates may be issued by the Forensic Medicine Organization. DHDs receive mortality data from five different sources including district hospitals, cemeteries, the Forensic Medicine Organization, service delivery points of DHD, and complementary sources made aware of any deaths, such as the clergy and voluntary health workers. Using a software package specifically designed for this purpose, data are compiled and checked for duplicate entries at the district level and then sent to the province center on a monthly basis. In this survey, the mortality data registered in 2006 and 2010 were used for analysis.

In 1990, the WHO produced a series of life tables for all member states. Since then the WHO has regularly updated and revised them for each member state to take account of new data on levels of child and adult mortality, including life expectancy at birth and the probability of dying according to gender [14]. These life tables have several uses and form the basis of all the WHO's estimates of mortality patterns worldwide [15]. They provide estimates of total deaths by age and gender that underpin WHO mortality and cause of death analyses [14]. We used the life table for the Islamic Republic of Iran developed by the WHO in 2009 [14] to determine reference age.

To calculate YPLL, the dataset was categorized into 5-year age groups based on each person's age at death. Then the age groups were subtracted from the relevant reference age using the following equation:

YPLL=Σ(N_i_^m^×L_i_^m^+N_i_^f^×L_i_^f^)

where N_i_^m^ (N_i_^f^) was the number of deaths of male (female) in age group; *i* multiplied by the standard life expectancy; L_i_^m^ (L_i_^f^) of male (female) at the age at which death had occurred. The YPLL for each individual were then added together to yield the total YPLL for all individuals in the population who died in a particular year. Finally, we calculated the YPLL for all sex- and age- groups by cause of mortality and reported these as percentages. All analyses were performed using the Stata version 11.0 (StataCorp, College Station, TX, USA).

## RESULTS

We identified 19,289 deaths; 503 deaths were excluded from analysis, 495 because age was not specified and eight because gender was not specified. Of 18,786 deaths, 9,127 occurred in 2006 and 9,659 in 2010. The details of the dataset are shown in Table 1.

**Figure 1:**
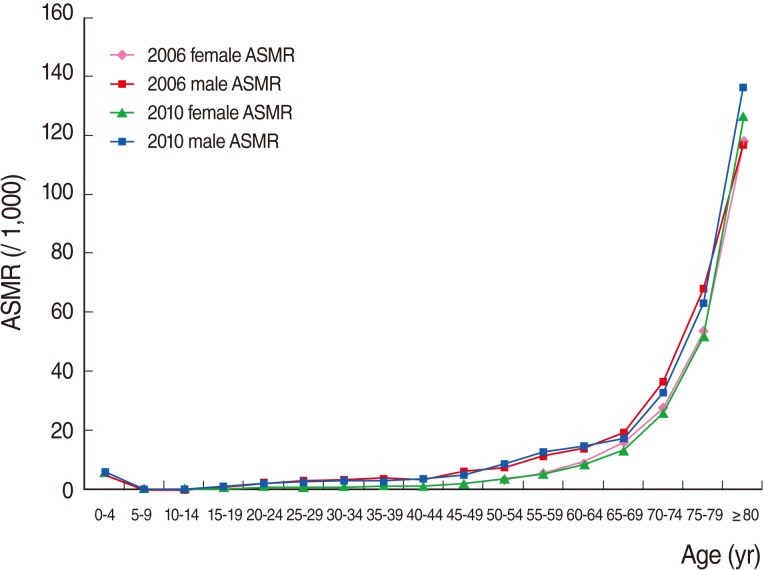
Age-specific mortality rate (ASMR) per 1,000 by year and sex, Hamadan Province, Iran 2006 and 2010.

Figure 1 shows age-specific mortality rates by gender in 2006 and 2010. It is apparent that the mortality rates were high for those aged less than five years and then declined rapidly in subsequent years. Mortality rates remained at essentially the same level from the ages of five to fifty. They then began increasing gradually in line with age and peaked dramatically after 70 years of age. Mortality rates were higher in men than women for all age groups both in 2006 and 2010. In addition, age-specific mortality rates in both genders for all age groups were nearly the same in 2006 and 2010.

**Figure 2:**
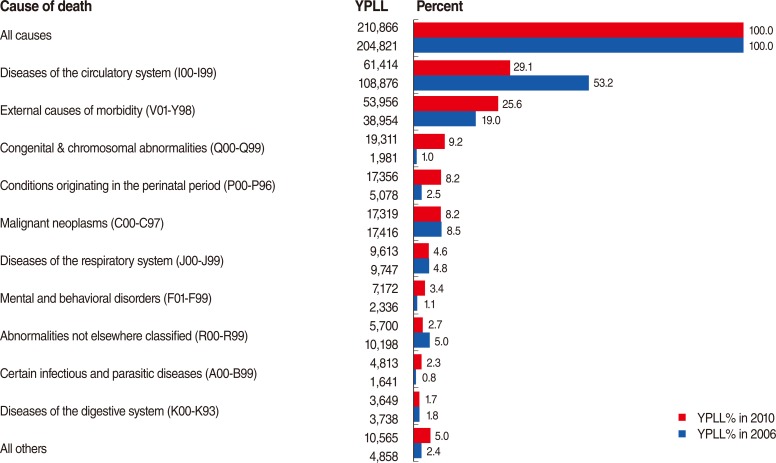
Years of potential life lost (YPLL) for all ages, Hamadan Province, 2006 and 2010.

Figure 2 shows the YPLL in Hamadan Province for all age groups in 2006 and 2010. The top bar shows the total percentage of YPLL from all causes (100%), and the bars below show the individual percentage of YPLL for each leading cause of death. We see that the greatest sources of YPLL, in both 2006 and 2010, were due to diseases of the circulatory system and external causes of morbidity. Although the percentage of YPLL from diseases of the circulatory system decreased dramatically in 2010 compared to 2006, the percentage of YPLL from external causes of morbidity increased significantly in 2010 compared to 2006.

**Figure 3:**
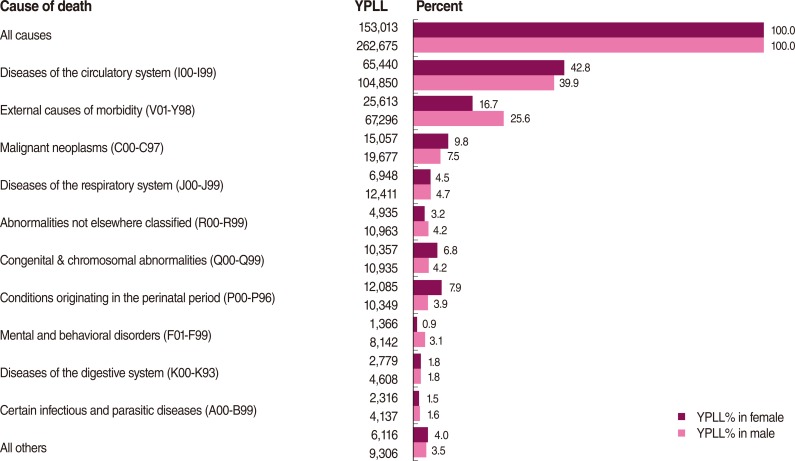
Years of potential life lost (YPLL) by sex, Hamadan Province, 2006 and 2010.

Figure 3 shows a similar presentation for YPLL in Hamadan Province for all age groups by gender. Diseases of the circulatory system and external causes of morbidity were the greatest sources of YPLL in both genders. The percentage of YPLL from diseases of the circulatory system was slightly greater in women than in men. However, the percentage of YPLL from external causes of morbidity was significantly greater in men than in women.

**Figure 4:**
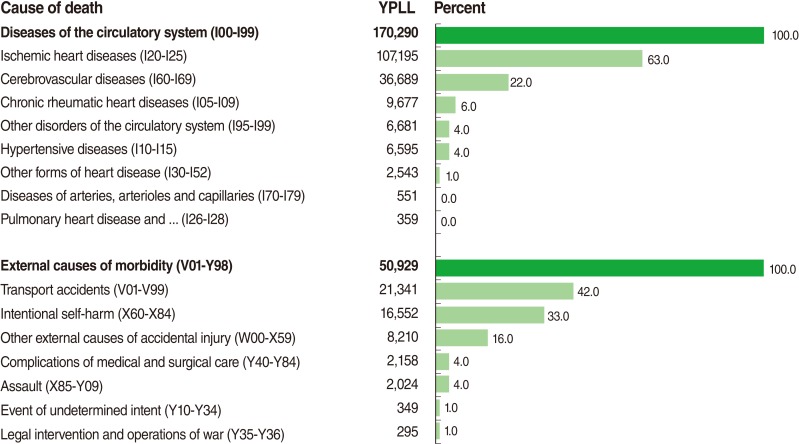
Years of potential life lost (YPLL) from the two greatest sources of YPLL including 'diseases of the circulatory system' and 'external causes of morbidity' by subgroup, Hamadan Province, 2006 and 2010.

Figure 4 shows the subgroups of the two greatest sources of YPLL. This shows that the percentage of YPLL from ischemic heart diseases and cerebrovascular diseases accounts for 85% of all percentages of YPLL from diseases of the circulatory system. In addition, the percentage of YPLL from transport accidents and intentional self-harm comprises 75% of all percentages of YPLL from external causes of morbidity.

## DISCUSSION

We have indicated that diseases of the circulatory system and external causes of morbidity were the first and second leading causes of premature mortality respectively in both 2006 and 2010. However, the percentage of YPLL due to diseases of the circulatory system decreased dramatically in 2010 compared to 2006, while the percentage of YPLL due to external causes of morbidity, congenital and chromosomal abnormalities and conditions originating in the perinatal period increased dramatically in 2010 compared to 2006. According to our findings, both the percentage and absolute number of YPLL due to diseases of the circulatory system decreased in 2010 compared to 2006, while those of external causes of morbidity (i.e. both the percentage and absolute number of YPLL) increased in 2010 compared to 2006. Hence, the decreasing trend of YPLL due to diseases of the circulatory system in 2010 compared to 2006 and the increasing trend of YPLL due to external causes of mortality in 2010 compared to 2006 appear to be unambiguous, although the percentages might be affected by variations in other causes of death. This issue indicates a changing pattern in non-communicable diseases from 2006 to 2010.

Another important finding of this survey was that the eight major causes of premature death were non-communicable diseases, suggesting an epidemiological transition of diseases from communicable to non-communicable. A population-based survey which was carried out recently in Hamadan Province indicated a silent progressive epidemic of chronic diseases that may lead to an increasing growth in non-communicable diseases in the near future [16]. Cigarette smoking, low physical activity, overweight and/or obesity, impaired fasting glucose, hypercholesterolemia, and hypertension were among the most common prognostic factors of non-communicable diseases which were reported in this survey.

According to our findings, the greatest sources of YPLL in both 2006 and 2010 were diseases of the circulatory system and external causes of morbidity respectively. Conversely, a similar survey [12] which was conducted in Iran in 2003 revealed that traffic injuries and diseases of the circulatory system were respectively the first and second leading causes of premature mortality. The results of another survey [11] which was carried out in Iran in 2009 support our findings. According to the results of this survey, ischemic heart diseases and transport accidents were respectively the first two leading causes of death in Iran. These findings imply a changing pattern in non-communicable diseases in recent years.

Age- and sex-specific mortality rates express mortality in quantitative terms for a certain age group in men or in women. Our results indicated an increasing trend of mortality rate with age. However, the trend of mortality rate was nearly the same in 2006 compared to 2010. In addition, the age- and sex-specific mortality rates, as well as the percentage of YPLL, were higher in men than women for all age groups. One reason that may explain this difference is that the frequency of external causes of morbidity, including transport accidents and intentional self-harm, are more common in men than in women. Furthermore, unhealthy life styles such as cigarette smoking, which may increase the probability of mortality, are more common in men than in women [16,17].

Today, the increasing growth in chronic diseases is becoming a serious threat to health and hence a major health challenge worldwide [18]. Our findings support this evidence. A similar survey [12] reported that the health and disease profile in Iran has made a transition from the dominance of communicable diseases to that of non-communicable diseases, as was shown in our results. In addition, there is a considerable body of other evidence indicating that chronic diseases play an important role in premature mortality [19-22]. A study which was carried out in Korea revealed that premature mortality due to transport accidents increased from 1991 to 1996 and then decreased from 2000 to 2006. On the other hand, premature mortality due to suicide increased gradually. The study showed that injuries caused major social and economic losses in Korea [23]. A survey in Turkey indicated that YPLL due to road traffic deaths were much greater than those due to common chronic diseases such as respiratory tract illnesses and diabetes mellitus [24]. Another survey, conducted in Saudi Arabia, showed an increasing trend in premature deaths due to road traffic accidents, particularly in males [25]. Such evidence suggests an epidemiological transition from communicable diseases to non-communicable diseases in developing countries.

This study had a number of limitations. We excluded 503 out of 19,289 deaths from analysis due to missing data. Although the number of missing data was small in comparison with the total number of deaths, this might introduce a negligible selection bias in the results. In addition, measuring YPLL requires reliable sources of mortality data. We used mortality data recorded in the death registry of District Health Network. The quality and accuracy of the mortality data depends primarily on the quality of the recorded death certification and we were unable to verify the accuracy of the death certificate data, thus potentially resulting in information bias. Another limitation of this study was that the YPLL employed in this survey were strongly influenced by death rates, especially premature death at younger ages, but was were not influenced by years of life lost due to time lived in less than perfect health. Other indicators of health status, such as disability-adjusted life years, combine information not only on the duration of life, but also on non-fatal disabling outcomes, and may encompass mortality as well as morbidity outcomes in a single summary measure. Nonetheless, the major strengths of YPLL are that the measure is simple to compute and interpret and it clearly indicates the deaths of younger people, unlike the usual measures of mortality rates which are dominated by the deaths of the elderly. Furthermore, YPLL represents a first step in estimating the overall burden of disease, taking into account premature death rates [4].

Despite the above limitations, the current survey may have a number of implications for health care policy. First, the major leading causes of YPLL in the target population, particularly the role of non-communicable diseases in premature mortality, were evident. Second, it was indicated that there has been a changing pattern in non-communicable diseases in recent years. The evidence provided by this study revealed the major sources of premature deaths in Hamadan Province, distinguishing it from other provinces in the country. Therefore, the results of the present study may be helpful for policymakers who plan preventive programs and prioritize risk factors in order to reduce the burden of non-communicable diseases.

The results of this survey revealed that the eight major causes of premature death were non-communicable diseases in both 2006 and 2010, especially ischemic heart diseases, cerebrovascular diseases, transport accidents, and intentional self-harm. Furthermore, our findings indicate a change in the role of non-communicable diseases in premature mortality in recent years.

## Figures and Tables

**Table 1:**
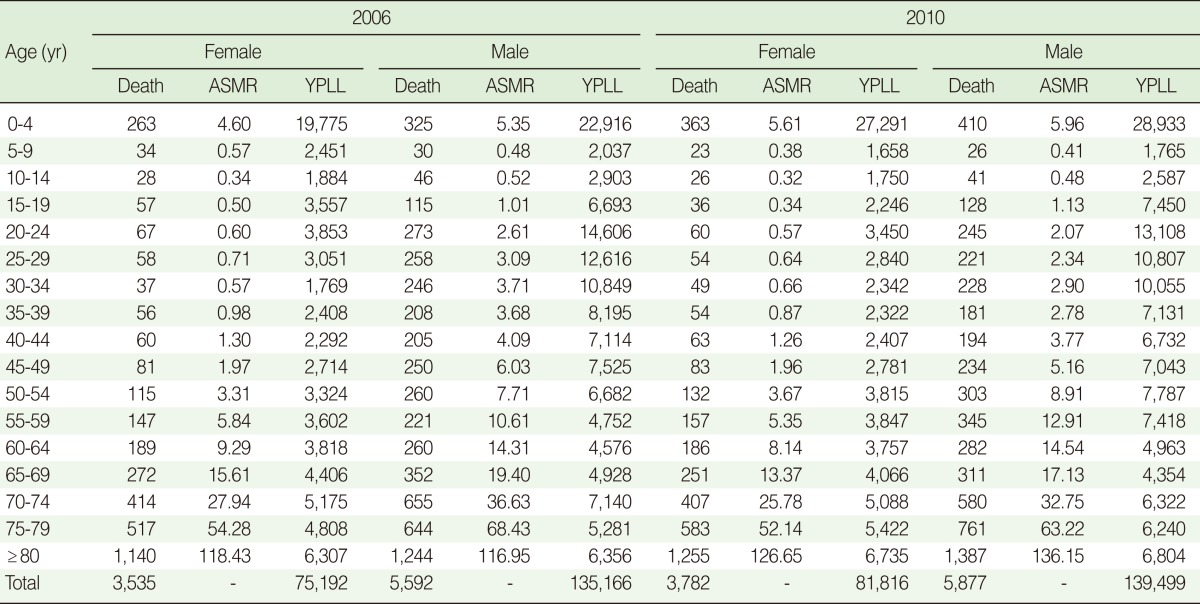
Distribution of deaths, age-specific mortality rates per 1,000 (ASMR), and years of potential life lost (YPLL) by year, gender, and age group
